# Genetic- and Fiber-Diet-Mediated Changes in Antibiotic Resistance Genes in Pig Colon Contents and Feces and Their Driving Factors

**DOI:** 10.3390/microorganisms11102370

**Published:** 2023-09-22

**Authors:** Tao Wang, Yuheng Luo, Xiangfeng Kong, Bing Yu, Ping Zheng, Zhiqing Huang, Xiangbing Mao, Jie Yu, Junqiu Luo, Hui Yan, Jun He

**Affiliations:** 1 Institute of Animal Nutrition, Sichuan Agricultural University, Chengdu 611130, China; 2 Key Laboratory of Animal Disease-Resistant Nutrition, Chengdu 611130, China; 3 Institute of Subtropical Agriculture, Chinese Academy of Sciences, Changsha 410125, China

**Keywords:** antibiotic resistance genes (ARGs), pig, high fiber, colon contents, feces

## Abstract

Comprehensive studies on the effects of genetics and fiber diets on antibiotic resistance genes (ARGs) remain scarce. In this study, we analyzed the profiles of ARGs in colonic contents and fecal samples of Taoyuan, Duroc, and Xiangcun pigs (n = 10) fed at different fiber levels. Through macrogenomic analysis, we identified a total of 850 unique types of ARGs and classified them into 111 drug resistance classes. The abundance of partially drug-resistant ARGs was higher in the colonic contents of local pig breeds under a large-scale farming model. ARGs were found to be widely distributed among a variety of bacteria, predominantly in the phyla *Firmicutes*, *Proteobacteria*, and *Bacteroidetes*. Fiber diets reduce the abundance of ARGs in colonic contents and feces, and mobile genetic elements (MGEs) and short-chain fatty acids (SCFAs) are important drivers in mediating the effect of fiber diets on the abundance of ARGs. In vitro fermentation experiments confirmed that butyric acid significantly reduced the abundance of ARGs. In summary, the results of this study enhanced our understanding of the distribution and composition of ARGs in the colon of different breeds of pigs and revealed that a fiber diet can reduce ARGs in feces through its Butyric acid, providing reference data for environmental safety.

## 1. Introduction

Antibiotics are commonly utilized in animal production to treat illnesses, combat infestation by pathogenic microorganisms, and enhance growth performance [1]. As a result of the widespread use of antibiotics, ARGs are enriched in the gut contents and feces of livestock and poultry, which can adversely affect host health [1]. ARGs are a type of functional gene carried by microorganisms that enable them to resist various antibiotics [2]. ARGs can be exerted through various mechanisms of their action, including reducing antibiotic permeability, modifying or replacing antibiotic targets, or facilitating antibiotic efflux [3]. For example, the *sul* gene in *Salmonella* resists sulfonamide antibiotics through antibiotic target substitution [4]. In addition, it was found that the *acrB* gene encodes a major multidrug exporting protein, and the *marB* gene, a transcription factor that down-regulates membrane permeability, confers resistance to a wide range of antibiotics in *E. coli* [5]. ARGs, as an emerging environmental pollutant, have attracted extensive attention worldwide. The WHO has recognized the antimicrobial resistance crisis as one of the most pressing threats to modern health care [6].

Significant differences exist in the gut microbiota of local breeds and commercial pigs in China due to differences in feeding practices and disease resistance [7]. Consequently, the composition or abundance of ARGs carried by microorganisms may also differ depending on the feeding pattern and diet of pigs [7]. For example, the abundance of ARGs was significantly higher in commercially farm-raised pigs than in semi-grazing-raised pigs due to differences in stocking densities, environments, and antibiotic use [7]. In addition, the physicochemical factors and bacterial composition in the intestinal contents and feces of livestock are directly affected by the physiological metabolism of the animals. It has been found that cold stress induces changes in the metabolite creatinine and oxyuranol in pigs, which in turn reduces the abundance of ARGs in colon contents and feces [8]. Numerous studies have shown that the abundance of drug-resistant bacteria and ARGs in the environment and crops increases when manure from antibiotic-exposed animals is used as fertilizer [9]. Importantly, there is growing evidence that ARGs can be transmitted to human gut microorganisms through a variety of pathways, such as the environment, aerosols, drinking water, and food-borne illnesses from animals [10]. Such transmission can directly expose humans to antibiotic-resistant pathogens, leading to potentially reduced efficacy of clinical antibiotic use and prolonged duration of infection. Thus, this transmission poses a significant threat to both animal and human health [11].

The Taoyuan pig is one of the famous local breeds in China [12]. The Duroc pig is a famous contemporary lean pork breed, originating from the United States. The Xiangcun pig is a cross between Taoyuan and Duroc pigs, which inherits the high-quality meat of Taoyuan pigs and also has excellent reproductive performance, roughness tolerance, and disease resistance [13]. However, little is known about the distribution of ARGs in the intestinal contents and feces of Taoyuan and Xiangcun crossbred pigs. In this study, we investigated the effects of different fiber levels on the differences in the composition and distribution of ARGs in the colonic contents and feces of different pig breeds (Taoyuan, Xiangcun, and Duroc) as well as the relationship of ARGs with host microorganisms and SCFAs using techniques such as macro-genomics, qPCR, liquid chromatography, and in vitro fermentation modeling, which provides reference data for animal breeding and management of environmental pollution.

## 2. Materials and Methods

### 2.1. Ethics Statement

The management of animal experiments involved in the research shall refer to the “Regulations on the Administration of Laboratory Animals” (Ministry of Science and Technology, China, revised in June 2004). The sample collection was approved by the Institutional Animal Care and Use Committee of Sichuan Agricultural University, Sichuan, China (No. 20181105).

### 2.2. Animal Trial and Sample Collection

The experiment selected, at 60 days of age, Taoyuan pigs (average weight: 13.87 ± 0.58 kg; purchased from Xiangcun Hi-Tech Agriculture Co., Ltd., Shaoyang, China), Duroc pigs (average weight 18.50 ± 1.09 kg; purchased from Linli Tianxin Seed Industry Co., Ltd., Zhuzhou, China), and their cross-bred varieties Xiangcun pigs (average weight 14.47 ± 0.15 kg; purchased from Xiangcun Hi-Tech Agriculture Co., Ltd.), and the experiment adopted a 3 × 2 factorial design, that is, 3 varieties of pigs (20 pigs for each variety, 10 pigs in each treatment group, and 60 pigs in total) were fed a high-fiber diet (crude fiber: 6–7%; digestible energy: 3.5%; and crude protein: 19.16%.) and a low-fiber diet (crude fiber: 2–3%; digestible energy: 3.49%; and crude protein: 19.15%). Wheat bran fiber was purchased from Chengdu Tubaite Technology Co., Ltd., Chengdu, China (manufacturer: JRS, model: WF200, and purity > 95%). All pigs were housed in single pens, each pen was equipped with feeders and nipple drinkers, the room temperature was 28 °C, water and food were free, and the experimental period was 28 d. Pigs were slaughtered on the last day of the experiment, and colonic contents were collected.

### 2.3. DNA Extraction, Library Construction, and Metagenomic Sequencing

Total genomic DNA was extracted from colon content samples using the E.Z.N.A.^®^ Soil DNA Kit (Omega Bio-tek, Norcross, GA, USA) according to the manufacturer’s instructions. The concentration and purity of extracted DNA were determined with TBS-380 and NanoDrop2000, respectively. DNA extract quality was checked on 1% agarose gel.

DNA extract was fragmented to an average size of about 400 bp using Covaris M220 (Gene Company Limited, Hong Kong, China) for paired-end library construction. Paired-end library was constructed using NEXTFLEX Rapid DNA-Seq (Bioo Scientific, Austin, TX, USA). Adapters containing the full complement of sequencing primer hybridization sites were ligated to the blunt end of fragments. Paired-end sequencing was performed on Illumina NovaSeq/Hiseq Xten (Illumina Inc., San Diego, CA, USA) at Majorbio Bio-Pharm Technology Co., Ltd. (Shanghai, China) using NovaSeq Reagent Kits/HiSeq X Reagent Kits according to the manufacturer’s instructions (www.illumina.com, accessed on 10 December 2021). Sequence data associated with this project were deposited in the NCBI Short Read Archive database (Accession Number: PRJNA849732).

### 2.4. Sequence Quality Control and Genome Assembly

The data were analyzed on the free online platform Majorbio Cloud Platform (www.majorbio.com, accessed on 10 December 2021). Briefly, the paired-end Illumina reads were trimmed of adaptors, and low-quality reads (length < 50 bp or with a quality value < 20 or having N bases) were removed with fastp [14] (https://github.com/OpenGene/fastp, version 0.20.0, accessed on 10 December 2021).

Metagenomics data were assembled using MEGAHIT [15] (https://github.com/voutcn/megahit, version 1.1.2, accessed on 10 December 2021), which makes use of succinct de Bruijn graphs. Contigs with a length ≥ 300 bp were selected as the final assembling result, then the contigs were used for further gene prediction and annotation.

### 2.5. Gene Prediction, Taxonomy, and Functional Annotation

Open reading frames (ORFs) from each assembled contig were predicted using Prodigal/MetaGene (http://metagene.cb.k.u-tokyo.ac.jp/, accessed on 10 December 2021). The predicted ORFs with a length ≥ 100 bp were retrieved and translated into amino acid sequences using the NCBI translation table (http://www.ncbi.nlm.nih.gov/Taxonomy/taxonomyhome.html/index.cgi?chapter=tgencodes#SG1, accessed on 10 December 2021).

A non-redundant gene catalog was constructed using CD-HIT [16] (http://www.bioinformatics.org/cd-hit/, version 4.6.1, accessed on 10 December 2021) with 90% sequence identity and 90% coverage. High-quality reads were aligned to the non-redundant gene catalogs to calculate gene abundance with 95% identity using SOAPaligner (http://soap.genomics.org.cn/, version 2.21, accessed on 10 December 2021).

Antibiotic resistance annotation was conducted using Diamond (http://www.diamondsearch.org/index.php, version 0.8.35, accessed on 10 December 2021) against the CARD database (https://card.mcmaster.ca/home, accessed on 10 December 2021) with an e-value cutoff of 1 × 10^−5^.

### 2.6. SCFA Determination

Sample pretreatment of colonic chyme was performed according to laboratory methods [17]. SCFA concentrations in the colon were determined using a gas chromatography system (CP-3800 GC, Varian, Inc., Walnut Creek, CA, USA) and following the method of Franklin et al. (2002) [18].

### 2.7. qPCR

DNA was extracted from feces, cecum contents, and cecum mucosa samples according to the instructions of the Stool Genomic DNA Extraction Kit (D2700, Solarbio, Beijing, China). The concentration and integrity of the DNA samples were determined using a Qubit Ultra Microspectrophotometer (NanoDrop 2000, Thermo Scientific, Waltham, MA, USA) and Gel Imaging (GelDocXR, Bio-Rad, Hercules, CA, USA) to detect the concentration, purity, and integrity of DNA samples. The abundance of 23 genes in the fecal samples was examined using qPCR (QS6FX, ABI, Waltham, MA, USA) (Table S4). Twenty-three genes included pairs of seven tetracyclines (tetA, tetC, tetM, tetW, tetQ, tetO, and tetX), one aminoglycoside (strB), two chloramphenicol analogs (cmlA and fexA), two sulfonamides (sul1 and sul2), two β-lactams (blaNDM and blaTEM), two macrolides (ermB and ermC), four (flor)/(chloror)/(am) phenolics (FCAs) (qnrS, oqxA, oqxA, and oqxB), and one mucilage (mcr-1) as well as intI1 and 16S rRNA genes for resistance. Absolute abundance of ARGs was calculated according to [8]. Normalized abundance of ARGs was calculated according to Yang et al. (2021) [8].

### 2.8. In Vitro Fermentation Assay

In vitro fermentation experiments were performed according to existing methods [19]. Duroc pigs were not fed antibiotics or other drugs for one month prior to feces collection and were in normal body condition. The collected feces were strongly mixed under aseptic conditions using 32% (wt/vol) inoculum (fecal slurry) in phosphate buffer, adjusted to pH 7.0 with HCl 0.1 M. and filtered through 4 layers of gauze. The filtrate bubbled with N_2_. Next, 8 mL of fermentation medium, 2 mL of fecal inoculum, and treatments were added to each 50 mL fermentation tube under anaerobic (N_2_) and aseptic conditions. The fermentation tubes were then incubated at 120 rpm for 20 h at 37 °C. After 20 h of fermentation, fermentation was stopped using a 15 min immersion in ice water, followed by collection of the fermentation broth, which was utilized for qPCR analysis of ARGs and MGEs. Peptone solution: 15 g of peptone (A505247, Sangon Biotech, Shanghai, China) was dissolved in about 1 L of anaerobic water, and the pH was adjusted to 7.0, then water was added to make 1 L of peptone solution. Reducing solution: 312 mg of cysteine (A600132, Sangon Biotech, Shanghai, China) and 312 mg of sodium sulfide (gz001, Knowles, Chengdu, China) were dissolved in 2 mL of 1 m of NaOH to prepare a reducing solution, which was adjusted to 50 mL with milliq water; Resazurin solution: a 0.1% (wt/vol) solution of Resazurin (A606726, Sangon Biotech, Shanghai, China) was prepared; and final fermentation medium: 1 L of peptone solution was mixed with 50 mL of reducing agent solution and 1.25 mL of Resazurin per liter of fermentation medium. Based on the content of SCFAs in the feces of the high-fiber diet group, we determined the final concentrations of AA, PA, and BA to be added in the in vitro fermentation, which were 70 μMol/mL, 30 μMol/mL, and 20 μMol/mL, respectively. 

### 2.9. Statistics

Data preprocessing was performed using Excel 2019 (Microsoft, Washington, DC, USA), and data statistics were performed using SPSS 22.0 (IBM Corp, New York, NY, USA). Graphical display of results was performed using GraphPad Prism 8. RPKM value was used for heat map data, Z-score was used for data standardization, and average clustering was used for cluster analysis. Correlation analysis used Spearman. The “protest” function in the vegan package was used to analyze the Procrustes correlation between the bacteriome and the resistome. Principal coordinate analysis (PCoA) and NMDS analyses were performed using the pure vegetation package in R software (R version 4.0.2), using ARG and normalized abundance values of bacterial communities with a Bray–Curtis distance algorithm. The distribution of ARGs in bacteria of different taxonomic levels was plotted as a Sankey diagram using the networkD3 package (https://cran.r-project.org/web/packages/networkD3, accessed on 15 August 2022) in R (v3.6.2). The mulberry graph is drawn using the plotly package in the R software. Circos and LEfSe analysis was produced and analyzed using the tools of the Megi Bio cloud platform. Redundancy analysis (RDA) was performed using Canoco 5.0 software.

RPKM (Reads Per Kilobase Million):

$$RPKM_{i}=\frac{R_{i}*10^{6}}{L_{i}*\sum_{1}^{n}\left(R_{j}\right)}$$

*R_i_* represents the abundance value of Genei in a sample, that is, the number of reads aligned to Genei in the sample; *L_i_* represents the nucleotide length of Genei; and $\sum_{1}^{n}\left(R_{j}\right)$ represents the sum of the reads corresponding to all genes in the sample.

Experimental design using a 3 × 2 design, Two-ANOVA was used to analyze two main effects and interaction effects. Results were expressed as means ± standard error, and *p* < 0.05 indicated a significant difference.

## 3. Results

### 3.1. Expression Profiling of ARGs in Colonic Contents of Different Pig Breeds

**Figure 1:**
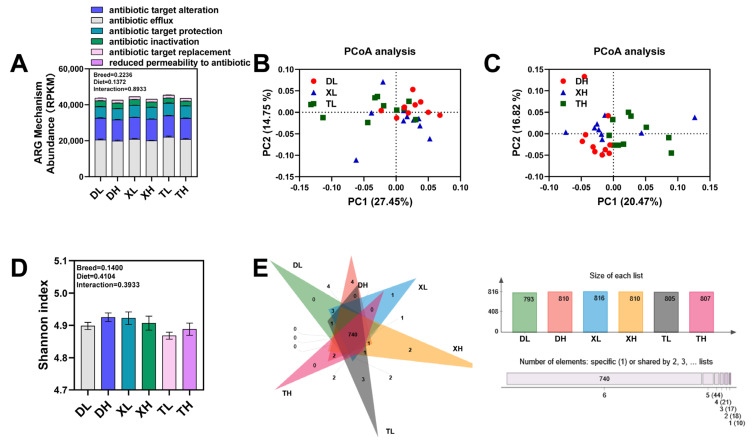
Comparison of relative abundance of ARGs (**A**). Plots of PCoA analysis between different breeds at low fiber level (**B**) and high fiber level (**C**). Comparison of the evenness (Shannon index) of ARGs (**D**). Venn diagram of ARGs (**E**); the bar graph on the right represents the number of ARG genes contained in different groups, and the horizontal bar graph represents the number of ARG genes shared by different groups. DL: Duroc pig group fed at low fiber level; DH: Duroc pig group fed at high fiber level; XL: Xiangcun pig group fed at low fiber level; XH: Xiangcun pig group fed at high fiber level; TL: Taoyuan pig group was fed at low fiber level; TH: Taoyuan pig group was fed at high fiber level, according to the mechanism of ARG action (antibiotic inactivation, antibiotic target alteration, antibiotic efflux, reduced permeability to antibiotic, antibiotic target replacement, and antibiotic target protection) classification.

**Figure 2:**
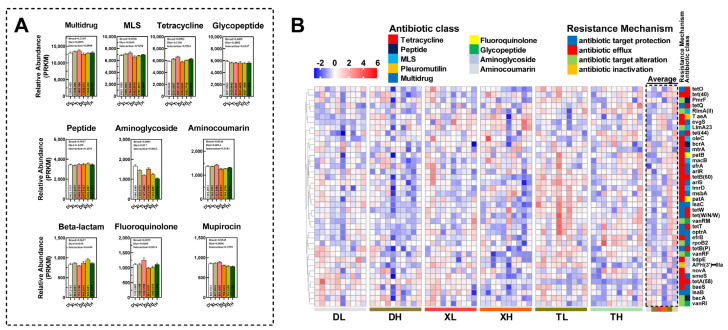
Comparison of relative abundance of drug resistance classification in Top 10 ARGs (**A**). Heatmap of enrichment analysis of ARGs of the top 40 genes in relative average abundance in different samples and groups (**B**). The different colors on the left side of the heatmap represent a different class of ARGs.

Deep macrogenomic sequencing of colon contents from Duroc, Taoyuan, and Xiangcun pigs fed at different fiber levels allowed us to cluster ORFs identified as genes coding for anti-microbial drug resistance proteins into 850 unique types of ARGs by comparison with the CARD database and could be further classified into 111 drug resistance classes (Table S1). Based on the categorization and statistical analysis of the resistance mechanisms of ARGs, it was determined that the main mechanisms by which pathogenic microorganisms in the porcine intestine develop antibiotic resistance are antibiotic efflux, antibiotic target alteration, and antibiotic target protection. No differences were found in the total relative abundance of ARGs in the colon of different pig breeds (Figure 1A and Table S2). However, the relative abundance of antibiotic efflux and antibiotic target protection was significantly increased in Taoyuan pigs compared to Duroc pigs (Table S2). The PCoA analysis (Figure 1B,C) showed relatively low similarity in the abundance of resistance genes between Duroc and Taoyuan pigs. However, as a cross between Duroc and Taoyuan pigs, the ARGs in the colon of Xiangcun pigs tended to be a mixture of characteristics of the two parental breeds. Further, we examined the alpha diversity of ARGs and found no differences (Figure 1D). Interestingly, the number of resistance genes in the colon was higher in Taoyuan and Xiangcun pigs than in Duroc pigs (Figure 1E). The abundance of drug resistance classes in the intestines of different breeds of pigs varies somewhat due to differences in feeding patterns and antibiotic use. By counting the drug resistance classes in the Top 10, we observed inter-breed differences in the relative abundance of drug resistance classes such as MLS, tetracycline, aminocoumarin, fluoroquinolone, and β-lactam (*p* < 0.1), with the highest relative abundance in Taoyuan pigs (Figure 2A). However, we also noted that the relative abundance of Aminoglycoside-like ARGs in the colon of Taoyuan pigs was significantly lower than that of Duroc pigs (Figure 2A). To further assess the similarity of ARG distribution among different breeds of pigs, we performed NMDS analysis (Figure S1A,B), and the results were consistent with the PCoA analysis.

To gain a comprehensive understanding of the composition of ARGs in porcine colonic contents, we analyzed the top 40 most abundant ARGs (Figure 2A). Among different breeds of pigs, we found intergroup differences in 16 of the top 40 ARGs (*macB*, *msbA*, *oleC*, *patA*, *lmrD*, *tetW*, *tetQ*, *bacA*, *tet (W/N/W)*, *RlMA (II)*, *tet (40)*, *efrB*, *lsaC*, *APH (3′)-IIIa*, and *PmrF*) (Figure 2B and Table S3). Interestingly, 14 of these 16 resistance genes had a higher average abundance in the intestine of Taoyuan pigs than that of Duroc pigs. In addition, the intestinal abundance of Xiangcun pigs was higher than that of Duroc pigs but lower than that of Taoyuan pigs.

**Figure 3:**
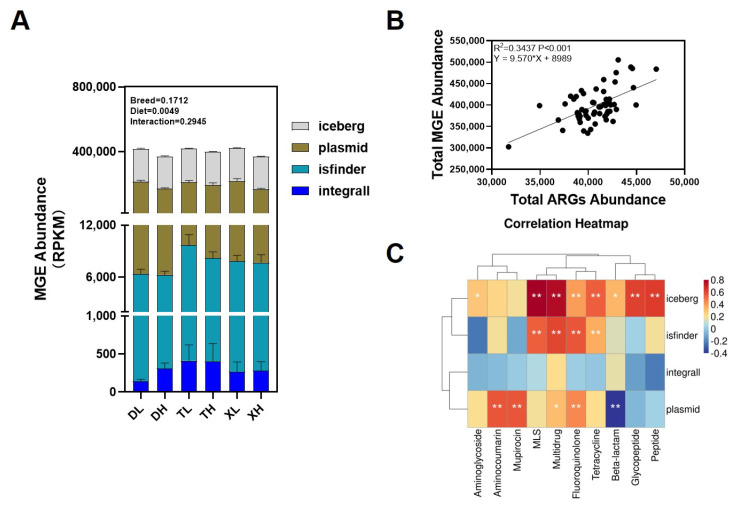
Abundance of different MGEs in pigs’ colon contents (**A**). Linear regression analysis of the relative abundance of ARGs and MGE in colon contents (**B**). Correlation analysis of drug resistance classes and MGE classification relative abundance of ARGs in colon contents (**C**). MGEs are classified as integral, iceberg, isfinder, and plasma. *: *p* < 0.05; **: *p* < 0.01. The color change represents the R value.

ARGs can be transmitted through the environment and diet, mainly through MGEs (iceberg, integral, equivocal, and plasma). Notably, we found no significant difference in the abundance of MGEs in the colon between different breeds of pigs (Figure 3A).

### 3.2. Fiber Diet Mediated Variation in the Profiling of Intestinal ARGs

Diet and metabolism have significant effects on the abundance and distribution of ARGs in the animal intestine. In our study, we investigated the effect of dietary fiber on ARGs in the colon of different breeds of pigs. We observed that high-fiber diets resulted in a significant reduction in the relative abundance of ARGs associated with altered antibiotic targets (Figure 1A and Table S2). Interestingly, the number of ARGs in the colon was higher in both Duroc and Taoyuan pigs in the high-fiber diet group than in the low-fiber diet group (Figure 1D). After classifying ARGs according to resistance to different antibiotic classes, we found that the relative abundance of ARGs resistant to Multidrug, MLS, Aminoglycoside, Aminocoumarin, Fluoroquinolone, Beta-lactam, and Mupirocin was either significantly or partially reduced in the high-fiber group decreased (Figure 2A). These findings suggest that dietary fiber has a reducing effect on the seven most common antibiotic resistance class genes in the porcine intestine. Via PCoA analysis, we also found that the flora of the high-fiber diet group and the low-fiber diet group were significantly separated, which also suggests that the high-fiber diet altered the distribution and abundance of ARGs in the porcine colon (Figure S2). After analyzing ARGs in the porcine colon at different fiber levels, we found that the high-fiber diet significantly reduced the mean relative abundance of 10 of the top 40 ARGs (*macB*, *efrA*, *arlS*, *optrA*, *patA*, *lmrD*, *novA*, *rpoB2*, *efrB*, and *kdpE*) (Figure 2B and Table S3).

We further compared the effects of different fiber levels on the abundance of MGEs in porcine colon. The results showed that high fiber levels significantly reduced the relative abundance of MGEs in the pig colon (Figure 3A), and this effect was consistent across different breeds of pigs. Furthermore, via correlation analysis, we found that the relative abundance of ARGs and MGEs showed a significant positive correlation (Figure 3B). Meanwhile, iceberg, isfinder, and plasma all showed significant positive correlations with some antibiotic resistance class genes (Figure 3C).

Overall, high fiber reduced the relative abundance of seven antibiotic resistance class genes in the colon and significantly reduced the abundance of MGEs in the colon. And MGEs were shown to be an important factor in the spread and enrichment of ARGs.

### 3.3. Relationship between Bacterial Taxonomic Dynamics and Antibiotic Resistance

**Figure 4:**
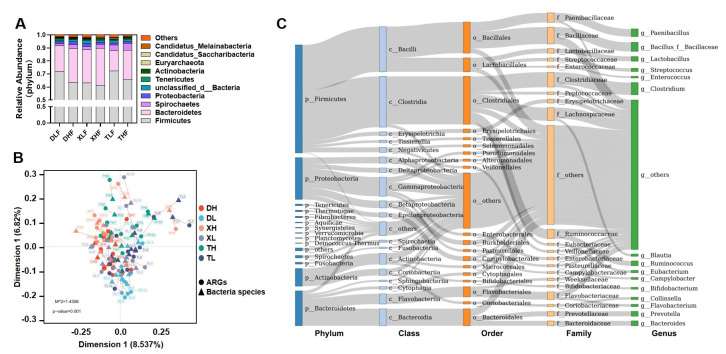
Relative proportions of microbes at different phyla levels in pigs’ colon contents (**A**). The association in the abundances between gut bacterial species and ARGs via Procrustes analyses. The dots show the ordination positions of the abundances of ARGs in each sample, and the triangulars indicate the ordination positions of the abundances of bacterial species. The length of lines between the dot and triangle shows the Procrustes residuals (**B**). (**C**) Distribution of 850 antibiotic resistance genes across different classes of bacteria (unclassified microorganisms were removed from the analysis). The colors of the rectangles represent the different classification levels. The length of the rectangle represents the number of ARGs.

ARGs present in the gut predominantly originate from microorganisms. Therefore, in order to explore the changes in the abundance and distribution of ARGs, it is crucial to understand the alterations in the bacteria from which these ARGs derive. Through metagenomic analysis, we identified that the dominant microbial phyla in the colonic contents of pigs were *Firmicutes*, *Bacteroidetes*, *Spirochaetes*, *Proteobacteria*, *Tenericutes*, *Actinobacteria*, and *Euryarchaeota*, accounting for more than 99% of the microbial composition (Figure 4A). The PCoA analysis of microorganisms revealed a distinct separation between Taoyuan pigs and Duroc pigs when they were fed a low-fiber diet. Conversely, Xiangcun pigs exhibited greater similarity to Taoyuan pigs. In the case of a high-fiber diet, there was no complete segregation among the different pig breeds (Figure S3A,B). These findings align with the results obtained from NMDS and PCoA analyses of ARGs, indicating a correlation between microbial abundance and ARGs. We further tested the correlation between ARGs and bacterial taxa using Procrustes analysis. Overall, the composition of the resistome was significantly correlated with the bacterial composition (m2 = 1.4356, *p* = 0.001) (Figure 4B). ARGs were found to be widely distributed across various bacteria, primarily in *Firmicutes* (mainly *Clostridia* and *Bacilli*), *Proteobacteria* (mainly *Gammaproteobacteria*), *Bacteroidetes* (mainly *Flavobacteriia* and *Bacteroidia*), and *Actinobacteria* (mainly *Actinobacteria* and *Coriobacteriia*) (Figure 4C).

**Figure 5:**
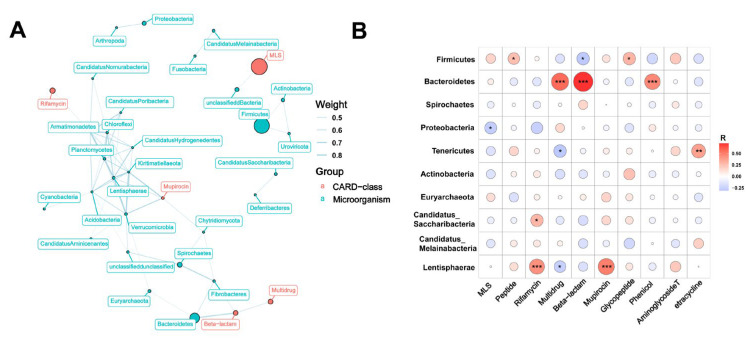
Intergroup correlation analysis between ARGs and microbes at the phylum level (**A**). Correlation analysis of ARGs and microbes at the phylum level (**B**). *: *p* < 0.05; **: *p* < 0.01; and ***: *p* < 0.001. The color change represents the R value.

To further investigate the relationship between microorganisms and resistance genes, we observed that the correlation weight between Firmicutes and Beta-lactam was determined to be -0.59 based on inter-group correlation analysis. Additionally, the weights between *Bacteroidetes* and Beta-lactam, *Verrucomicrobia*, *Kiritimatiellaeota*, and Mupirocin were all greater than 0.5 (Figure 5A). Correlation analysis revealed that both *Bacteroidetes* and *Firmicutes* exhibited significant correlations with more than two classes of ARGs (Figure 5B). These findings further support the notion that Bacteroidetes and Firmicutes act as the primary hosts of ARGs. Furthermore, when comparing the relative abundance of microbial phyla, it was observed that the average abundance of *Bacteroidetes* and *Firmicutes* in Taoyuan pigs was lower than that in Duroc pigs, providing further confirmation that Bacteroidetes and Firmicutes are indeed the main hosts of ARGs.

### 3.4. Effects of Breed and Fiber Diet on the Distribution of ARGs in Swine Feces and Their Potential Factors

**Figure 6:**
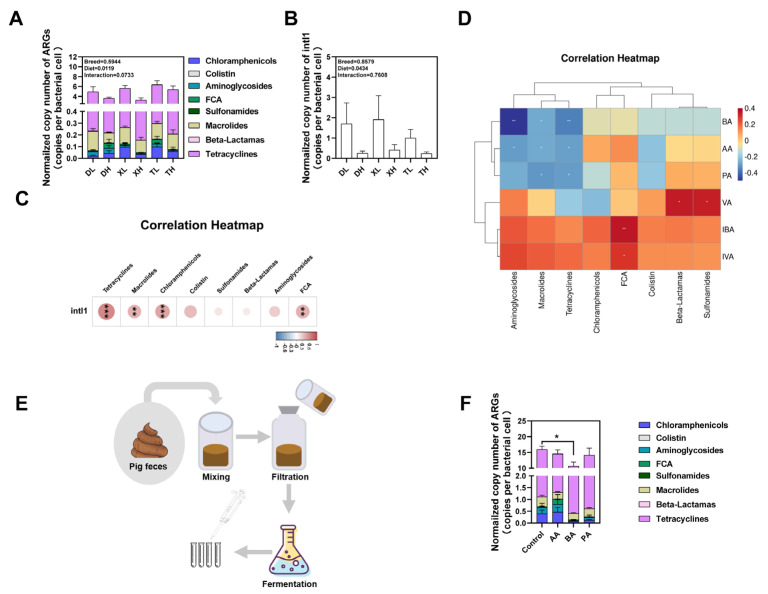
Normalized abundances of ARGs (**A**) and the intI1 gene (**B**) in fecal samples. (**C**) The correlation between ARGs and the intI1 gene in fecal samples. (**D**) The correlation between ARGs and the SCFAs in fecal samples. (**E**) In vitro fermentation mode diagram. (**F**) The influence of AA, BA, and PA on the ARGs profile. AA: Acetic acid, PA: Propionic acid, BA: Butyric acid, VA: Valeric acid, IPA: Isopropylic acid, IBA: Isobutyric acid, and IVA: Isovaleric acid. *: *p* < 0.05; **: *p* < 0.01; and ***: *p* < 0.001. The color change represents the R value.

We detected a total of 21 ARG subtypes via absolute quantification in the feces of different breeds of pigs. These ARGs conferred resistance to eight prominent classes of antibiotics, encompassing tetracyclines, aminoglycosides, chloramphenicol, sulfonamides, β-lactams, macrolides, fluoroquinolones, and mucins (Figure 6A). The outcomes revealed that the inclusion of a fiber diet led to a significant reduction in the normalized abundance of ARGs within swine feces, with no discernible variation in ARGs abundance noted between different breeds (Figure 6A). Additionally, we extended our analysis to the normalized abundance of the integrase gene intI1 and observed a consistent pattern: the high-fiber diet yielded a noteworthy reduction in the normalized abundance of the integrase gene *intI1* within feces, with no appreciable difference across breeds (Figure 6B). Correlation analysis was employed to delve further into these relationships. We discovered a significant positive correlation between the normalized abundance of intI1 and drug-resistant classes such as Tetracyclines, Macrolides, Chloramphenicols, and Fluoroquinolones (Figure 6C). This finding substantiates the notion that MGEs likely constitute a pivotal factor influencing the spectrum of ARGs present within feces.

When the body’s physiological metabolism changes, the relevant metabolic molecules are secreted into the intestinal tract, affecting the composition of the bacterial community in the intestinal tract, which in turn affects the composition of ARGs. We examined SCFAs, the main products of fiber metabolism, in feces using liquid chromatography and found that a high-fiber diet significantly increased the levels of AA, PA, and BA in the feces of different pigs (Figure S4). Furthermore, our correlation analysis disclosed that BA, AA, and PA exhibited statistically significant negative correlations with the three ARGs bearing the highest relative abundances (Figure 6D). These observations collectively imply that our high-fiber dietary intervention might effectively modulate the excretion of ARGs within fecal matter through the intermediary influence of its metabolic byproducts, the SCFAs. To substantiate the connection between SCFAs and ARGs, we proceeded to assess the effects of BA, AA, and PA upon ARGs borne by microorganisms following the fermentation of fecal flora using an in vitro fermentation model (Figure 6E). The outcomes demonstrated that BA exerted a substantial reduction in the normalized abundance of ARGs within swine fecal fermentation broth (Figure 6F). On the whole, our findings suggest that fiber diets wield the capacity to influence the excretion of ARGs within distinct pig breeds through the intermediary action of their metabolic product, BA. Furthermore, this action extends to the suppression of ARG dissemination by diminishing the presence of MGEs within fecal matter.

## 4. Discussion

The distribution and abundance of ARGs are dependent on many factors such as the environment, diet, genetics, and species [20,21]. The purpose of this study was to investigate the effects of breeds, hybrid genetics, and diet in different regions on the distribution, abundance, and transmission of ARGs.

The abundance and structure of ARGs in the colonic contents of different breeds of pigs varied, and the hybrid breed Xiangcun pigs partially acquired the characteristics of ARGs in the colon of their parents. This phenomenon can be attributed to the fact that the intestinal microbial communities of local pig breeds are subjected to antibiotic selective pressure during large-scale breeding, resulting in the acquisition of a diverse range of antibiotic resistance genes and that this process contributes to a rapid increase in the abundance levels of resistance genes [22,23]. This explains why the abundance of ARGs was higher in local pig breeds such as Taoyuan pigs under the same feeding pattern. Of interest, when categorizing the ARGs of different breeds, we found that the average number of ARGs was significantly higher in Taoyuan and Xiangcun pigs than in Duroc pigs. This finding is consistent with the results of a previous study, which revealed that Tibetan pigs exhibited a higher abundance and number of ARGs than Duroc pigs under the same standard feeding conditions [7]. Furthermore, it has been demonstrated that the gut microbial community of the parental generation can influence the microbial structure of the offspring through intergenerational inheritance [24]. Considering that the hosts of ARGs in the gut are mainly microorganisms, this also explains why the colonic ARGs profiles of the crossbred pig breeds showed a mixture of Duroc and Taoyuan pig characteristics. In general, most classes of ARGs in swine feces are those that are resistant to macrolide, peptide, tetracycline, and nitroimidazole. These resistance classes of genes are more common in swine farms worldwide, including those in China, the United States, and Europe, and are used to treat infections with pathogens such as *E. coli*, *Streptococcus*, and *Staphylococcus aureus* [25,26,27]. This can be partly explained by the fact that these classes of antibiotics have been used in pig production for a long time, despite geographic and breed differences.

Dietary fiber-enriched components presented the effect of decreasing the levels of ARGs to some extent and significantly reduced the relative abundance of MGEs. It has been shown that metabolic changes significantly affect the distribution and number of ARGs in animals [8]. Dietary fiber has been proven to play an important role in the physiological states of energy metabolism, lipid metabolism, and glucose metabolism in animals [28,29]. The results of the present study indicate that a high-fiber dietary regimen was effective in reducing the abundance of specific drug-resistant classes of ARGs in the pig colon. This finding further suggests that dietary fiber improves the antibiotic resistance environment in the animal gut by improving intestinal metabolism, decreasing the number of ARGs, and adjusting their distribution. Dietary fiber intake is closely related to gut health status and helps promote the maintenance of gut microbial diversity [30]. The finding that Duroc and Taoyuan pigs within the high-fiber diet group had relatively more species of ARGs in the colon at different fiber levels also side-steps the previous observation. The implementation of the fiber diet significantly reduced the level of MGEs present in the colon, and furthermore, the relative abundance of MGEs was significantly and positively correlated with the content of ARGs. The level of propagation of ARGs was mainly achieved through the synergistic action of multiple MGEs, including insertion sequences, transposons, and gene cassettes/integrators, either within microbial DNA molecules or between DNA molecules [31]. Plasmids and phages play an important role in facilitating the spread of ARGs among bacteria. Numerous studies have consistently demonstrated that pathogenic bacteria acquire and evolve ARGs via MGEs. For instance, research has provided evidence that *E. coli* frequently undergoes the transmission of ARGs through MGEs during periods of mass colonization in inflammatory conditions [32]. Clinical cases have also observed the horizontal transfer of ARG plasmids between *Klebsiella pneumoniae* and *E. coli* [33]. In summary, ARGs carried by the intestinal flora can be transferred and enriched by MGEs, and this transfer of ARGs further facilitates the colonization and proliferation of pathogenic bacteria and other organisms within the host’s intestine.

The microbiota in the colon carries a wide range of ARGs. Among them, microorganisms of the genus *Clostridium* carry the highest number of ARGs. It has been shown that *Clostridium* spp. microorganisms carry a significant number of ARGs in their populations and exhibit multiple drug resistance in a variety of environments including *Clostridium difficile*, *Clostridium perfringens*, and *Clostridium capillarum* [34,35]. It is interesting to note that a significant number of ARGs were also detected in microorganisms of the genus *Lactobacillus*, a finding that suggests the need for caution in the development of potential *Lactobacilli* in dairy production to prevent potential antibiotic resistance problems. Different dietary structures and additives can all have an effect on the structure of the intestinal flora of pigs. It has been shown that the abundance of *Bacteroidetes* and *Turicibacter* in the cecum and jejunum significantly increases with increasing the proportion of fiber in the feed and significantly increases the diversity of the flora [36]. Silage, on the other hand, was found to significantly elevate the relative abundance of *Proteobacteria* in the cecum [37]. Alternatives to antibiotics, such as probiotics, have also exhibited the capacity to enhance microbial community structure and augment the prevalence of beneficial bacteria, including *Clostridium*, *Lactobacillus*, and *Turicibacter*, among others [38]. Although, fiber diet, silage, and probiotics can all improve the intestinal flora structure of pigs, which in turn has an impact on the excretion of ARGs in feces. However, considering the economics, ease of handling, and potential value of partially replacing protein feeds, fiber diets may be of higher value in reducing fecal ARGs.

Dietary fiber results in a significant reduction in the abundance of ARGs carried within fecal matter, accomplished through the actions of their metabolites. The expulsion of animal feces ensues as a consequence of the digestion and metabolic processes of ingested food within the intestines, subsequently undergoing excretion from the organism’s body. Alterations in the metabolic processes within the animal’s intestines may exert influence upon fecal attributes [39], which may include the abundance of ARGs [8]. Consequently, the observed impact of a fiber-rich diet on the abundance of ARGs within feces can be attributed to the actions of fiber-derived metabolites within the intestinal milieu. Consistent with previous studies, our findings also showed that a fiber diet significantly increased the abundance of SCFAs in feces [40]. In addition, there was a significant positive correlation between butyric acid (BA), acetic acid (AA), and propionic acid (PA) as well as the abundance of some drug-resistant classes of ARGs, suggesting that a high-fiber diet may be influencing the excretion of ARGs in the feces through their metabolites, SCFAs. In addition, we demonstrated through validation in an in vitro fermentation model that BA significantly reduced the abundance of ARGs. As an acidifying agent, BA is widely used in practice to improve pig performance, increase feed utilization, and reduce feed-to-weight ratio, as well as to lower the pH of pig feces and reduce the total number of colonies and *Escherichia coli* counts, thereby reducing the pathogenic microbial load of nursery pigs [41,42]. These results also demonstrated that BA may achieve the effect of reducing ARGs by inhibiting the proliferation of host microorganisms of ARGs such as pathogenic microorganisms in feces. In addition, through the successful application in a fermentation model, we were also able to recognize that BA can realize its potential to promote environmental safety by being added to agricultural production processes such as composting.

## 5. Conclusions

In this study, we examined how genetics and dietary fiber affect the distribution and composition of ARGs in the colonic contents and feces of pigs. Our findings revealed that in a large-scale farming environment, the Chinese native pig breed Taoyuan exhibited a higher abundance of ARGs when compared to commercial Duroc pigs, and their respective ARG profiles exhibited significant differences. The intracolonic ARG profile of the hybrid pig breed, the Xiangcun pig, displayed a composite characteristic originating from both Taoyuan and Duroc pig lines. This discovery underscores the significant impact of strain variation on ARG profiles and highlights the need for future research efforts aimed at elucidating the dynamics of ARG profiles in crossbred breeds. However, it is essential to incorporate larger and more diverse sample sizes for a more comprehensive perspective on ARG propagation and changes during breeding. Moreover, our study revealed that high-fiber diets can partially mitigate the abundance of ARGs in colonic contents and feces. This reduction can be attributed to MGEs and short-chain fatty acids, primarily butyric acid (BA), which play pivotal roles in mediating these changes. Given the widespread use of fiber diets and their metabolite, BA, in practical livestock production, they hold significance in mitigating the risk associated with ARGs during real-world production. These findings suggest the potential of high-fiber diets and antibiotic alternatives, such as acidifiers, in reducing the prevalence of antibiotic resistance genes in farm animal sources. Nevertheless, for a more comprehensive exploration of the impact of dietary structure on ARGs in animal sources, larger sample sizes are required to compare ARG dynamics across different regions and various feeding technologies. In summary, this study provides valuable insights that can inform the optimization of animal breeding strategies and deepen our understanding of strategies for mitigating environmental risks associated with antibiotic resistance genes.

## Data Availability

Supporting data are available from the authors if requested.

## Supplementary Materials

The following supporting information can be downloaded at https://www.mdpi.com/article/10.3390/microorganisms11102370/s1, Figure S1: Plots of NMDS analysis between different varieties at low fiber level (A) and high fiber level (B); Figure S2: Principal component analysis of ARGs gene abundance between different fiber levels; Figure S3: Plots of PCA analysis of microorganisms at different levels for different varieties at low fiber levels (A) and high fiber levels (B); Figure S4: Changes in SCFAs content in feces of different pig breeds at different fiber levels. The content of AA (A), PA (B), BA (C), VA (D), IVA (E), and IBA (F); Table S1. Information of 850 unique antimicrobial resistance genes (ARGs); Table S2. Effect of fiber level on relative abundance of ARGs mechanism in colon of different pig breeds; Table S3. Effect of fiber level on relative abundance of top 40 ARGs in colonic content of different pig breeds; Table S4. Primers used in this study.
