# Supplementary material for: Genetic- and Fiber-Diet-Mediated Changes in Antibiotic Resistance Genes in Pig Colon Contents and Feces and Their Driving Factors
Source: Microorganisms. 2023 Sep 22;11(10):2370. doi: 10.3390/microorganisms11102370 (PMC10609257; doi:10.3390/microorganisms11102370)
Supplement: Supplementary file 1 [file microorganisms-11-02370-s001.zip › Supplement file.pdf]

Genetic and fiber diet-mediated changes in antibiotic resistance genes in pig colon contents and feces and their driving factors

Tao Wang[1,2†], Yuheng Luo[1,2†], Xiangfeng Kong[3*], Bing Yu[1,2], Ping Zheng[1,2], Zhiqing Huang[1,2], Xiangbing Mao[1,2], Jie Yu[1,2], Junqiu Luo[1,2], Hui Yan[1,2], Jun He[1,2*]

1Institute of Animal Nutrition, Sichuan Agricultural University, Chengdu, Sichuan 611130, P. R. China

2Key Laboratory of Animal Disease-resistant Nutrition, Chengdu, Sichuan 611130, P. R. China

3Institute of Subtropical Agriculture, Chinese Academy of Sciences, Changsha, Hunan 611130, P. R. China 410125, P. R. China

*Corresponding author: Institute of Animal Nutrition, Sichuan Agricultural University, Chengdu, Sichuan 611130, P. R. China; Tel: +86-13419354223, Fax: +86-28-86291781, Email: hejun8067@sicau.edu.cn; nnkxf@isa.ac.cn.

† Contribute equally to this work.

Keywords: Antibiotic resistance genes (ARGs); pig; High fiber; colon contents; Feces

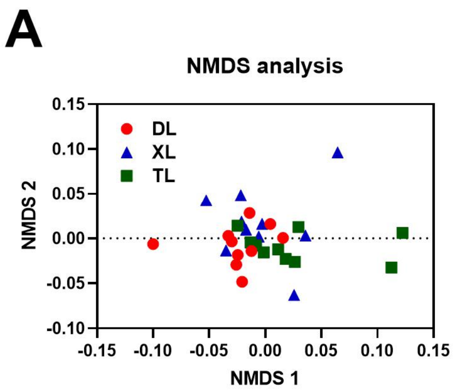

Figure S1: Plots of NMDS analysis between different varieties at low fiber level (A) and high fiber level (B).

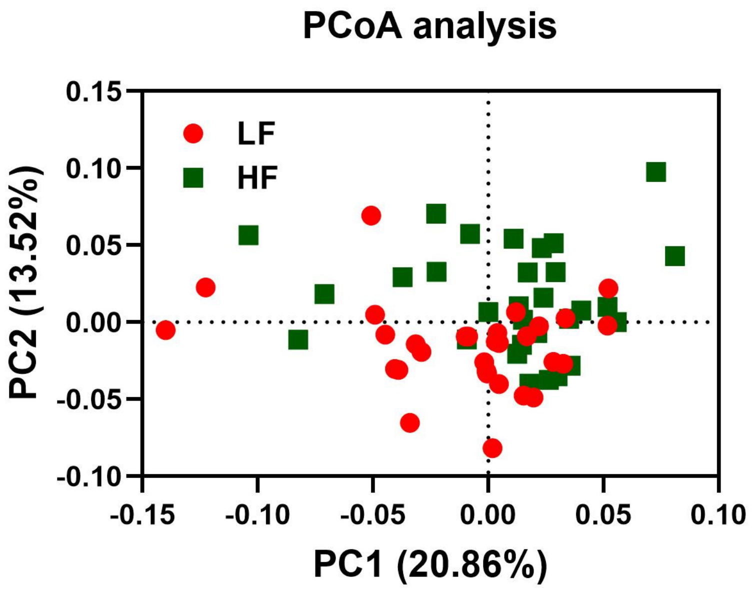

Figure S2: Principal component analysis of ARGs gene abundance between different fiber levels.

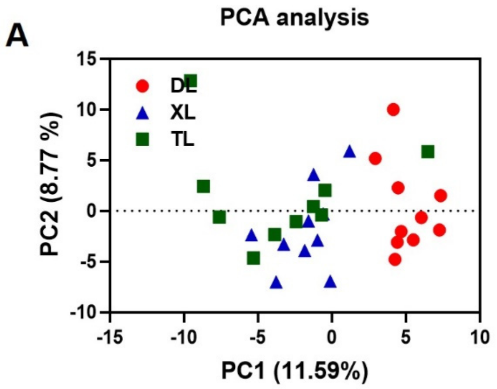

Figure S3: Plots of PCA analysis of microorganisms at different levels for different varieties at low fiber levels (A) and high fiber levels (B).

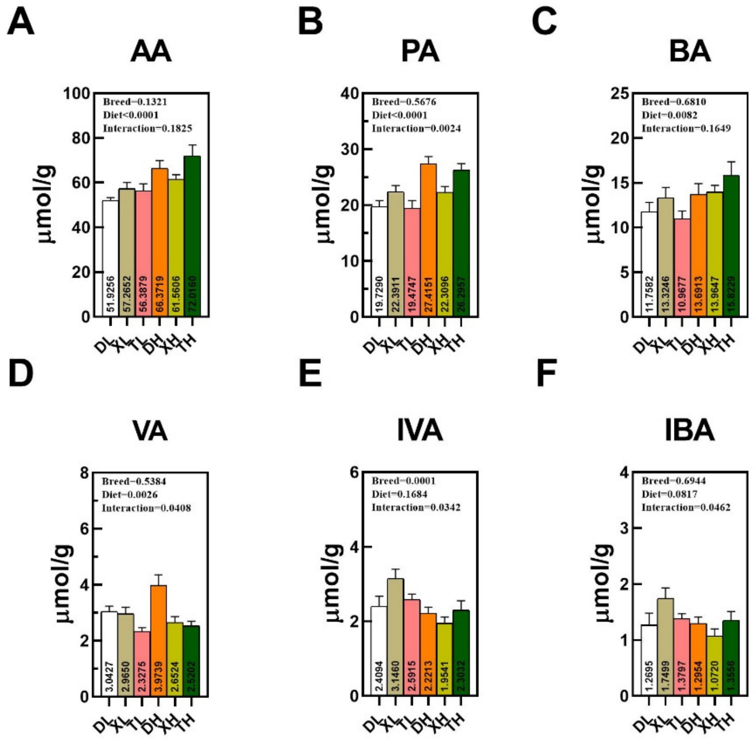

Figure S4: Changes in SCFAs content in feces of different pig breeds at different fiber levels. The content of AA (A), PA (B), BA (C), VA (D), IVA (E), and IBA (F).
